# Return to preinjury pivoting sports after anterior cruciate ligament reconstruction is different between males and females, as are the patient‐reported reasons

**DOI:** 10.1002/ksa.12588

**Published:** 2025-01-16

**Authors:** Jay R. Ebert, Liza Kneebone, Peter Edwards, Ross Radic, Peter D'Alessandro

**Affiliations:** ^1^ School of Human Sciences (Exercise and Sport Science) University of Western Australia Perth Western Australia Australia; ^2^ HFRC Perth Western Australia Australia; ^3^ Orthopaedic Research Foundation of Western Australia Perth Western Australia Australia; ^4^ Perth Orthopaedic & Sports Medicine Research Institute Perth Western Australia Australia; ^5^ School of Allied Health Curtin University Perth Western Australia Australia; ^6^ Perth Orthopaedic & Sports Medicine Centre Perth Western Australia Australia; ^7^ Department of Orthopaedics Royal Perth Hospital Perth Western Australia Australia; ^8^ School of Medicine and Surgery University of Western Australia Perth Western Australia Australia; ^9^ Fiona Stanley and Fremantle Hospitals Group South Metropolitan Health Service Perth Western Australia Australia

**Keywords:** anterior cruciate ligament reconstruction, meniscal repair, return to sport, sex

## Abstract

**Purpose:**

To investigate return to sport (RTS) after anterior cruciate ligament reconstruction (ACLR), differences based on sex and concomitant meniscal repair, and identify reasons why patients do not RTS.

**Methods:**

Overall, 232 patients undergoing ACLR, with or without concomitant meniscal repair, that were actively participating in pivoting sports at the time of injury, were prospectively recruited. At 2 years, return to preinjury pivoting sport was investigated and, if they had returned, whether they felt their performance was at (or better) or below preinjury status. Specific reasons for not returning were identified. RTS rates and reasons for not returning were compared based on sex and meniscal repair.

**Results:**

Overall, 140 patients (60.3%) had returned to their preinjury pivoting sport, of which 98 (70.0%) felt they were performing at (or beyond) preinjury status. While a significantly greater (*p* = 0.024) percentage of males (66.9%) versus females (52.4%) had returned to pivoting sports by 2 years, no differences (*p* = 0.708) were seen based on concomitant meniscal repair. Overall, 92 patients (39.7%) had not RTS, with primary reasons being loss of interest (21.7%), too busy due to their work and/or family environment (22.8%), or fear of reinjury or lacking confidence (17.4%). Other less‐reported reasons included ongoing knee issues (6.5%) or not feeling physically ready (5.4%).

**Conclusions:**

This study outlined specific reasons why community‐level patients do not RTS, with RTS status (and reasons for not returning to preinjury pivoting sports) differing between males and females, with the latter returning at a significantly lower rate overall.

**Level of Evidence:**

Level IV.

**Abbreviations**
ACL: anterior cruciate ligament
ACLR: anterior cruciate ligament reconstruction
RTS: return to sport

## INTRODUCTION

Anterior cruciate ligament (ACL) ruptures are common [23], with surgical ACL reconstruction (ACLR) being the standard treatment [25], especially for those looking to return to pivoting sports. While return to sport (RTS) is often a primary goal for patients after ACLR, reported RTS rates continue to be relatively low with only 65% of patients returning to their preinjury level of sport and 55% to competitive level sport after ACLR [1]. Despite these reported rates, most patients undergoing ACLR expect to be able to return to their preinjury level of sport [29].

With the goal of improving RTS outcomes and reducing reinjury rates after ACLR, several guidelines have been published to assist clinicians working with these patients [4, 8, 15, 16, 18, 19, 31]. However, the reasons why patients do not RTS after ACLR are multifactorial and, as per a recent review [27], may include (though not limited to) surgical technique such as graft choice and augmentation, duration and quality of therapy, physical performance, psychological factors, patient‐related variables (such as age and sex) and the nature of the specific sport. Numerous recent studies have highlighted many of these factors associated with RTS after ACLR [2, 11, 14, 20, 21, 22, 26, 30]. However, while studies have sought to investigate the association between these factors and the ability of patients to RTS, limited studies exist that have identified actual patient‐reported reasons for not returning [3, 13, 28]. A recent systematic review reported RTS rates specifically in females [7], though suggested that future studies should determine specific reasons for not returning to sport.

This study sought to (1) investigate the RTS rates, patient‐perceived sporting performance level, differences based on sex and concomitant meniscal repair in community‐level ACLR patients and (2) understand patient‐reported reasons for not returning to preinjury pivoting sports after ACLR, along with reported differences based on sex.

## MATERIALS AND METHODS

### Patient sample and recruitment

A total of 276 patients undergoing primary ACLR with a hamstring autograft between June 2015 and June 2022 were prospectively recruited into an institutional research programme. Surgery was undertaken by one of three fellowship‐trained sports orthopaedic surgeons. Concomitant meniscal repair was undertaken in 142 patients (51.4%), while eight patients (2.9%) underwent concomitant lateral extra‐articular tenodesis (LET). Criteria for LET broadly aligned with the STABILITY trial [9], with those undergoing LET generally presenting with at least three of the following criteria: female, <25 years of age, seeking a return to pivoting sports, generalised ligamentous laxity, >5° hyperextension and a high‐grade pivot shift on clinical examination or examination under anaesthesia. The overall group was heterogeneous with respect to the mechanism of injury, including netball (*n* = 56), Australian Rules Football (*n* = 54), soccer (*n* = 49), basketball (*n* = 27), rugby (*n* = 18), skiing (*n* = 9), hockey (*n* = 9), fighting contact sports (*n* = 6), racquet sports (*n* = 3), during another sporting or recreational activity (*n* = 32) or as a result of a home‐ or work‐based incident (*n* = 12) or from a motor vehicle accident (*n* = 1). Ethics approval for recruitment into the institutional research programme was provided by the relevant hospital Human Research Ethics Committee and all patients provided consent prior to participation.

### Two‐year RTS status

**Figure 1:**
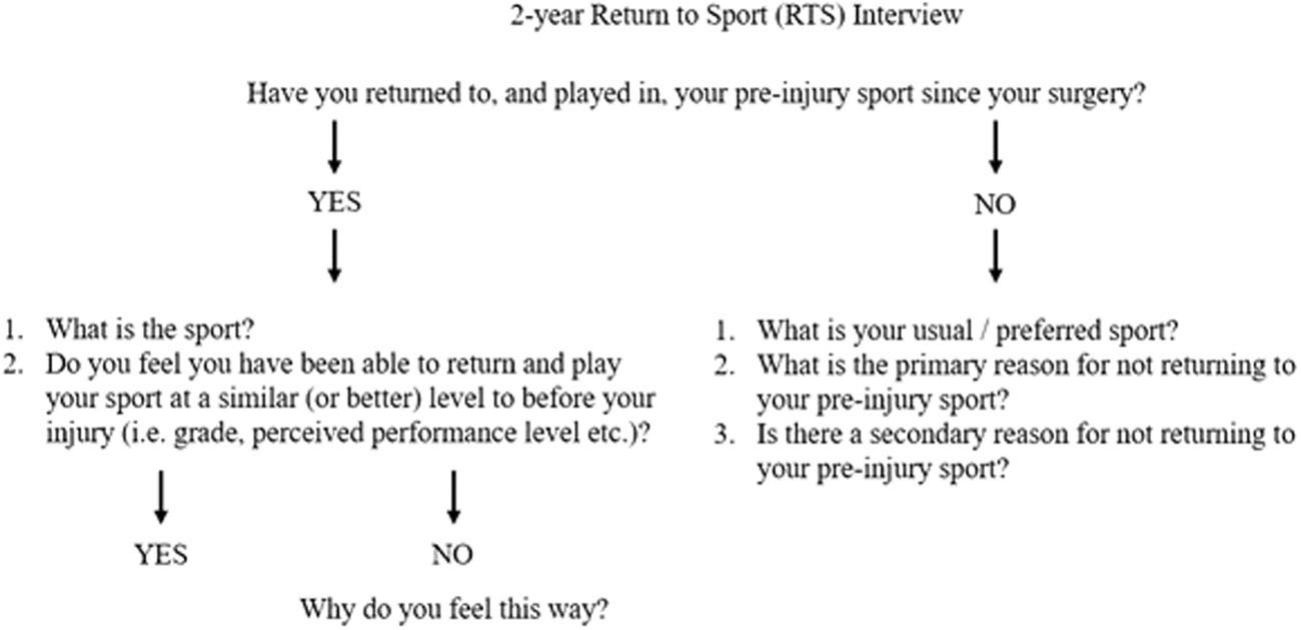
Semistructured questions asked in all patients at 2‐years postsurgery regarding their ability to return to sport within the first 2 years after their primary ACLR.

All patients were reviewed at 2‐years postsurgery, with respect to their ability to return to preinjury pivoting sports within the 2‐year postoperative period. A series of semistructured questions were posed to all patients (Figure 1), via a single interviewer, with responses from patients documented in writing and collated. The questions to be asked were originally drafted by co‐authors prior to the study onset. However, the specific questions (and wording of questions) were subsequently modified based on discussion with other experienced therapists, as well as feedback from a pilot clinical cohort of ACLR patients spanning 1–2‐years postsurgery that were not included in the cohort recruited as part of the current study. This ensured that the questions to be asked were clear and concise and were applicable and logical to the ACLR cohort before recruitment was initiated, and the questions were to be administered for those included in the current study. Initially, patients were asked whether they have (or had, in the presence of a reinjury) returned to their primary preinjury pivoting sport and, if so, whether they felt their performance was at (or beyond) their preinjury status. If they reported that their performance remained inferior, the reasons for feeling that way were documented. If patients had not returned to their primary preinjury pivoting sport within the first two postoperative years, the primary reason for not returning was documented. Patients were further given the option of reporting a secondary reason for not returning.

### Data and statistical analysis

First, the means (standard deviation, range) or *n* (%) of relevant demographics (age, body mass index, sex), injury and surgery characteristics (time from injury to surgery, injury mechanism, concomitant surgery status) were calculated and presented for the group overall (*n* = 276). However, of the 276 patients recruited, 44 (15.9%) reported they were not actively and/or regularly participating in pivoting sports prior to their injury, with no desire to return after their surgery. Therefore, these patients were omitted from the RTS analysis. Subsequently, the number (and percentage) of those that had, or had not, returned to preinjury pivoting sports were calculated, as was the number (and percentage) of those that reported they were (or were not) performing at their preinjury performance status. The number (and percentage) of males and females that had returned to preinjury pivoting sports within the first 2 years after surgery was calculated and statistically compared using *χ* ^2^ tests. This sex‐based comparison was also undertaken for those who had returned to sports that felt their performance was similar (or better) to preinjury status, versus those that felt it was inferior. Furthermore, the number (and percentage) of those that underwent concomitant meniscal repair (versus those that did not require meniscal repair) that had returned to preinjury pivoting sports within the first 2 years after surgery was calculated and statistically compared using *χ* ^2^ tests. This meniscal‐based comparison was also undertaken for those who had returned to sports that felt their performance was similar (or better) to preinjury status versus those that felt it was inferior. Finally, patient‐reported reasons for not returning to sport, or for returning to sport though why they felt their performance remained inferior to their preinjury status were collated and presented. These reasons were also tabulated according to sex. Where relevant, statistical analyses were conducted using the Statistical Package for the Social Sciences (SPSS) Version 29.0 (SPSS inc.). Statistical significance was determined at a *p* < 0.05.

## RESULTS

Patient demographics (age, body mass index, sex), injury (injured knee relative to dominant limb, time from injury to surgery, injury mechanism) and surgery characteristics (concomitant surgery) of the full cohort are shown in Table 1.

**Table 1:** Patient demographics, along with injury and surgery characteristics, of the full cohort (*n* = 276) recruited.

Variable	Measure	*n* = 276
Age (y)	Mean (SD)	28.2 (8.5)
	Range	16–50
Body mass index	Mean (SD)	25.7 (3.3)
	Range	18.0–39.8
Time injury to surgery (weeks)	Mean (SD)	10.8 (15.8)
	Range	1–150
Sex (males)	*n* (%)	149 (54.0%)
Injured knee is dominant limb	*n* (%)	126 (45.7%)
Injured activity		
Pivoting sports	*n* (%)	231 (83.7%)
Other nonpivoting sport/recreational activity	*n* (%)	32 (11.6%)
Other	*n* (%)	13 (4.7%)
Concomitant surgery		
Meniscus repair	*n* (%)	142 (51.4%)
Lateral tenodesis	*n* (%)	8 (2.9%)

Abbreviation: SD, standard deviation.

### Two‐year RTS status

Of the 232 patients that were regularly participating in pivoting sports prior to their injury, 140 (60.3%) had returned to their primary preinjury pivoting sport, of which 98 (70.0%) felt they were performing at (or beyond) their preinjury status (Table 2). A total of 42 patients (30.0%) felt their performance was below preinjury status for reasons that included feeling slower and/or less explosive running in straight lines and/or with change of direction (*n* = 38, 90.5%) or still lacking sport‐specific fitness and conditioning (*n* = 4, 9.5%) that subsequently affected their performance level.

**Table 2:** Return to sport status within 2 years of primary ACLR, including the perceived performance level in patients that had returned to their primary preinjury pivoting sport.

Question		Response	*n*	%
Returned to preinjury sport		No	92	39.7
		Yes	140	60.3
		Yes, at same perceived preinjury performance level	98	70.0
		Yes, below perceived preinjury performance level	42	30.0
Reported primary reason for not returning to preinjury pivoting sport within 2 years		Lost interest in primary pivoting sport	20	21.7
		Too busy to train and/or play (work/family reasons)	21	22.8
		Work responsibilities (cannot afford to reinjure)	8	8.7
		Fear of reinjury and/or lacking confidence in knee	16	17.4
		Not feeling physically ready	5	5.4
		Ongoing knee issues (i.e., pain, symptoms)	6	6.5
		Other health issues (not knee‐related)	2	2.2
		Season schedule	5	5.4
		Fell pregnant	6	6.5
		Other	3	3.3
Reported secondary reason for not returning to preinjury pivoting sport within 2 years		Lost interest in primary pivoting sport	1	1.1
		Too busy to train and/or play (work/family reasons)	6	6.5
		Work responsibilities (cannot afford to reinjure)	12	13.0
		Fear of reinjury and/or lacking confidence in knee	3	3.3
		Not feeling physically ready	5	5.4
		Ongoing knee issues (i.e., pain, symptoms)	1	1.1
		Other health issues (not knee‐related)	0	0.0
		Season schedule	0	0.0
		Fell pregnant	0	0.0
		Other	1	1.1

*Note*: Also shown are primary (and secondary) reasons for patients not returning to their primary preinjury pivoting sport.

Abbreviation: ACLR, anterior cruciate ligament reconstruction.

A significantly greater (*p* = 0.024) percentage of males (66.9%) versus females (52.4%) that were participating in pivoting sports prior to their injury had returned to pivoting sports by 2‐years postsurgery (Table 3). Of those that had RTS, no sex‐based difference (*p* = 0.790) was seen in those that did, or did not, perceive their performance to be on par (or better) than their preinjury status (Table 3). No difference (*p* = 0.708) in the 2‐year RTS rate was observed between those that did (or did not) undergo concomitant meniscal repair (Table 4). Similarly, no meniscal‐based difference (*p* = 0.557) was seen in those that did, or did not, perceive their performance to be on par (or better) than their preinjury status (Table 4).

**Table 3:** RTS status within 2 years of primary ACLR for males and females, including the perceived performance level in patients that had returned to their primary preinjury pivoting sport.

RTS status	Males (*n* = 127)	Females (*n* = 105)
RTS	85 (66.9%)	55 (52.4%)
RTS (at same preinjury performance level)	59 (69.4%)	37 (67.3%)
RTS (below preinjury performance level)	26 (30.6%)	18 (32.7%)
No RTS	42 (33.1%)	50 (47.6%)

Abbreviations: ACLR, anterior cruciate ligament reconstruction; RTS, return to sport.

**Table 4:** RTS status within 2 years of primary ACLR for those that did, or did not, undergo concomitant meniscal repair, including the perceived performance level in patients that had returned to their primary preinjury pivoting sport.

RTS status	Meniscal repair (*n* = 117)	No repair (*n* = 115)
RTS	72 (61.5%)	68 (59.1%)
RTS (at same preinjury performance level)	54 (75.0%)	48 (70.6%)
RTS (below preinjury performance level)	18 (25.0%)	20 (29.4%)
No RTS	45 (38.5%)	47 (40.9%)

Abbreviations: ACLR, anterior cruciate ligament reconstruction; RTS, return to sport.

Of the 232 patients that were regularly participating in pivoting sports prior to their injury, 92 (39.7%) had not returned to their primary preinjury pivoting sport (Table 2). The primary reasons for not returning are documented in Table 2. Of those that had not returned to pivoting sports, the main reasons for not returning included losing interest in their primary preinjury pivoting sport and no longer seeking to return (21.7%), being too busy to train, play or commit to their chosen sport due to a busy work and/or family environment (22.8%) or reasons associated with fear of reinjury or lacking confidence in the knee (17.4%) (Table 2). Other less‐reported reasons included ongoing knee issues (6.5%) or not feeling physically ready (5.4%), while six female patients (6.5%) had fallen pregnant within 2 years of surgery (Table 2). Of those that had not returned to their sport, 29 patients (31.5%) reported a secondary reason for not returning to their primary preinjury pivoting sport (Table 2), with most of these due to busy work/family commitments and/or not being able to afford reinjuring again (due to financial reasons and/or time off work).

### Reinjuries and reoperations

Of the 232 patients that underwent ACLR were regularly participating in pivoting sports prior to their injury and were reviewed at 2‐years postsurgery, several reinjuries and/or reoperations were observed over the 2‐year postoperative period. First, *n* = 9 patients (3.9%) had reruptured within the first 2 years, while *n* = 2 patients (0.9%) had ruptured their contralateral ACL, all of which had reinjured after returning to, and participating in, their primary preinjury pivoting sport. Of interest, 10 patients (of 11, 90.9%) that experienced ipsilateral retears or contralateral tears reported that they felt they were performing at (or better) than their preinjury status prior to their secondary injury. Patients that had experienced a retear or contralateral tear were included in the RTS analysis as they had indeed all returned to their preinjury pivoting sport within 2 years of their primary ACLR and were all confident reporting their perceived performance level prior to their reinjury.

Second, other significant reinjuries and/or reoperations included osteoarticular autograft transplantation (OATS, Arthrex) or Mosaicplasty (Smith & Nephew) (*n* = 5, all within 18–21 months postsurgery and while participating in their primary preinjury pivoting sport) and meniscal repair (*n* = 4, all within 11–13 months postsurgery, with *n* = 3 suspected to occur while playing or training for their primary preinjury pivoting sport, and *n* = 1 of unknown origin). All of these patients were retained in the RTS analysis. For those that underwent secondary OATS/Mosaicplasty, all patients had RTS, with *n* = 4 reporting that felt they were performing at (or better) than their preinjury status prior to their secondary injury, with *n* = 1 reporting an inferior performance level. For those that underwent secondary meniscal repair, *n* = 2 patients had RTS, of which *n* = 1 reporting that felt they were performing at (or better) than their preinjury status prior to their secondary injury, with *n* = 1 reporting an inferior performance level. The remaining *n* = 2 had not returned to pivoting sports, due to reasons that included ongoing knee issues (*n* = 1) and being too busy (*n* = 1).

Finally, several other patients underwent secondary procedures within 3–6 months (*n* = 3), 6–12 months (*n* = 6), 12–18 months (*n* = 0) and 18–24 months (*n* = 2) of their primary ACLR, for reasons that included manipulation under anaesthesia, arthroscopy for general chondral or scar tissue debridement, meniscectomy or notch debridement. For those that underwent a secondary procedure within 3–6 months, *n* = 1 had returned to pivoting sports at perceived inferior performance level, with *n* = 2 having not returned to sport (*n* = 1 fell pregnant, *n* = 1 for other reasons). For those that underwent a secondary procedure within 6–12 months, *n* = 2 had returned to pivoting sports and felt they were performing at (or better) than their preinjury status, with *n* = 4 having not returned to sport (*n* = 1 ongoing knee issues, *n* = 1 not being able to afford being off work again, *n* = 2 due to fear of reinjury). For those that underwent a secondary procedure within 18–24 months, *n* = 1 had returned to pivoting sports at a perceived inferior performance level, with *n* = 1 having not returned to sport and reporting they were too busy with work and family duties.

## DISCUSSION

The most important findings of the current study are that only 60% of patients had returned to pivoting sports within 2 years of undergoing ACLR, which was significantly greater in males than females. Furthermore, no difference in RTS rates were seen based on the concomitant need for meniscal repair. Finally, this study confirms the multifactorial nature of the reasons for patients failing to return to their preinjury sports after ACLR, primarily losing interest in their primary sport, being too busy to train, play, or commit due to a busy work and/or family environment or reasons associated with confidence and/or fear of reinjury. These reported reasons also differed based on sex.

In the current study and in patients that underwent ACLR and were regularly participating in pivoting sports prior to their ACL injury, 60.3% had returned to pivoting sports within 2 years. Despite the majority of patients undergoing ACLR expecting to be able to return to their preinjury level of sport [29], reported RTS rates are variable [1, 5, 12]. Ardern et al. [1] had previously reported that only 65% of patients return to their preinjury level of sport and 55% to competitive level sport after ACLR. This aligns with the 2‐year RTS rate in the current study, albeit this was almost 70% of males and only 52% of females. Of interest and specifically in female athletes, a recent systematic review reported that 69% of females returned to sport at an average of 10.8 months after ACLR [7]. However, most of the female cohorts included were at elite and subelite levels, as opposed to a wider community‐level cohort as was employed in the current study that would be more likely to be representative of the majority of ACLR. A recent study undertaken in patients undergoing ACLR with both hamstring and quadriceps tendon autografts reported greater Tegner activity scores in males (versus females) at 1‐ and 2‐years postsurgery, albeit the actual RTS rate was not reported [6]. In contrast to the current study which demonstrated no difference in the 2‐year RTS rate based on the presence of concomitant meniscal repair, other studies have reported that the presence of meniscal repair reduces and/or delays the RTS rate [2, 17, 24]. However, the current study grouped all patients with meniscal repair, irrespective of tear type, location, or size.

In patients that had RTS in the current study, 70% of patients reported they felt they were performing at (or better) than their preinjury status. While no differences in the perception of good (versus inferior) performance were observed between those that had returned based on sex or concomitant meniscal repair, overall, 30% of patients felt their performance level had not yet returned. As reported, reasons for this lack of performance level generally related to feeling slower and/or less explosive running in straight lines and/or with change of direction or still lacking sport‐specific fitness and conditioning that subsequently affected their performance level. While we acknowledge this is a very subjective outcome measure and the actual performance of patients could not be measured, let alone a comparison made to their preinjury status, this is something that has been rarely reported. Some studies have reported RTS rates and further asked patients whether they had returned to the same (or higher) level of sport [3, 28]. In a study by Toale et al. [28], patients reported whether they had returned to play, in which sport and to what level. If they had not returned, the reason for not returning was reported, with lack of confidence or concerns about reinjury (49%) and persistent postoperative impairments (39%) being the most reported reasons. More recently, Bashaireh et al. [3] reported on the use of a self‐administered questionnaire that asked athletes to answer whether they had returned to sports. If they had, they were asked to comment on whether this was their preinjury sports and whether it was the same frequency, duration and intensity. However, patients have not been specifically asked about their perceived performance, and why they felt their sporting performance remained inferior to preinjury status. Of interest, in the current study, all patients that experienced ipsilateral retears or contralateral tears had done so after returning to their preinjury pivoting sports, and all (but one) subsequently reported that they felt they were performing at (or better) than their preinjury status.

While several studies have reported an array of factors that are associated with RTS status (or not returning to sport) after ACLR [2, 11, 14, 20, 21, 22, 26, 30], this study sought to ascertain specific reasons from patients why they had not returned to sport. In the current study, the most common primary reasons included losing interest in their primary preinjury pivoting sport and no longer seeking to return (22%), along with being too busy to train, play, or commit to their chosen sport due to a busy work and/or family environment (23%). While most patients expect to be able to return to their preinjury level of sport prior to surgery [29], priorities after surgery can change. Of interest, these primary reasons differed between males and females, with males more commonly reporting being too busy, though females more commonly reporting a loss of interest. While a previous study reported that 30% of female footballers terminated their careers due to injury (as well as for other reasons such as not being able to reconcile their sport with work, losing motivation, or becoming a wife/mother) [10], Webster et al. [29] previously reported that being female was a significant predictor of changing postoperative expectations and giving up sport participation after ACLR.

Another common primary reason for not returning was fear of reinjury or lacking confidence in the knee (17%) and the association between psychological readiness to RTS and actual RTS has been previously reported [32]. However, in the limited studies that have reported the actual reasons why patients had not returned to sports, fear of reinjury or a lack of confidence in the knee have been reported more commonly. In a study by Toale et al. [28], 16% of patients had not RTS at 2 years, with the most common patient‐reported reasons including fear of reinjury (27.5%) and lack of confidence (19.4%). Bashaireh et al. [3] reported that 68% of patients that had not returned stated a lack of confidence or fear of reinjury. Finally, a study by Kiran et al. [13] employed an open‐ended question about what factors stopped patients from returning to sport or influenced the level of sport, with fear of reinjury reported by 79% of patients. In the current study, females (56%) versus males (44%) were also more likely to report fear of re‐injury or a lack of confidence in the knee as a primary reason for not returning to sport. Lower psychological readiness to RTS in females after ACLR has been recently reported [6]. Regardless, while it has been reported recently that greater psychological readiness in females after ACLR may be associated with a greater reinjury risk [33], identifying patients with a higher fear of reinjury and lower knee‐related level of confidence is important throughout the rehabilitation process and appropriate educational and counselling strategies adopted to address these issues.

Other less‐reported reasons included ongoing knee issues (7%) which was reported more commonly by males (67%) versus females (33%), as well as not feeling physically ready (5%), more commonly reported by females (60%) versus males (40%). Of interest, six females (which was 12% of the female cohort that not returned to sports) fell pregnant after their ACLR, though before they had returned to sports. To the best of our knowledge, this has not been previously reported and highlights the multifactorial nature of not returning to sport. This information is important in better interpreting the reported RTS rates and appreciating there are patient‐related reasons that extend beyond the more commonly reported assumptions such as ongoing knee issues, as well as a lack of physical and psychological readiness. As reported earlier, Toale et al. [28] reported that patients had not RTS at 2 years due to fear of reinjury (27.5%) or lack of confidence (19.4%), though other reasons included external life factors (16.6%), residual knee pain (10%) and subsequent injury (5%). In addition to a lack of confidence or fear of reinjury, Bashaireh et al. [3] reported that 39% of patients that had not returned reported ongoing knee impairments, albeit this study was undertaken in a cohort that was 98% male. Kiran et al. [13] also reported reasons such as persistent pain (20.9%) and instability (16.2%), as well as a lack of interest (28%).

The prospective nature of patient recruitment, relative balance of males and females, as well as the large patient cohort of community‐level ACLR patients, are strengths of the current study. However, this study is not without limitations. First, all patients underwent ACLR with a hamstring autograft (for which the outcomes, therefore, may lack generalisability across other graft types). Second, the 2‐year RTS rate was compared between those that did (or did not) undergo concomitant meniscal repair. While it was beyond the scope of the current study, and numbers across varied meniscal tear types may not permit a robust statistical comparison, detailed information on the types of meniscal tears and/or surgical procedures to address these meniscal tears was not collected. Therefore, a specific RTS subanalysis could not be provided based on the varied types of meniscal tears and/or surgical procedures. Nonetheless, the current study did not assess the timing of RTS, rather whether patients had (or had not) returned within 2 years, so the influence of the type of meniscal tear/repair may be less so within that full 2‐year timeframe. Third, these were community‐level patients, and undergoing varied out‐patient rehabilitation programmes, as opposed to elite and/or professional athletes. The age range of the entire group spanned 16–50 years, albeit all patients included in the RTS analysis were actively and regularly participating in pivoting sports at the time of their knee injury, and this study sought to investigate RTS and reasons for not returning to sport in a community‐level cohort commonly encountered in clinical practice. Future studies may seek a longer follow‐up period; however, this makes patient recall of preinjury performance status more challenging, to investigate longer‐term RTS rates and how these responses may change.

## CONCLUSION

The current study found that only 60% of patients had returned to pivoting sports within 2‐years of undergoing ACLR, which was significantly greater in males versus females. In those that had returned to sport, no sex‐based differences were seen in patient‐perceived sporting performance, while no differences were seen in RTS rates and/or perceived sporting performance based on concomitant meniscal repair. Importantly, this study has outlined specific reasons for why community‐level patients do not return to preinjury pivoting sports, highlighting the multifactorial nature of patient decision‐making. A better understanding of the reasons behind not returning to sport, and how these may differ based on sex, will assist in patient education and counselling, maintaining patient motivation and reducing barriers to returning to sports and individualising rehabilitation and RTS pathways.

## AUTHOR CONTRIBUTIONS

*Conceived and designed the study*: Jay R. Ebert, Peter Edwards, Ross Radic and Peter D'Alessandro. *Supervised the conduct of the study*: Jay R. Ebert, Peter Edwards and Liza Kneebone. *Analysed the data*: Jay R. Ebert, Peter Edwards and Liza Kneebone. *Wrote the initial drafts*: Jay R. Ebert and Peter Edwards. *Critically revised the manuscript*: Jay R. Ebert, Peter Edwards, Liza Kneebone, Ross Radic and Peter D'Alessandro. *Ensure the accuracy of the data and analysis*: Jay R. Ebert, Peter Edwards, Liza Kneebone, Ross Radic and Peter D'Alessandro. I confirm that all authors have seen and agree with the content of the manuscript and declare that the work has not been submitted or published elsewhere in whole or part.

## CONFLICT OF INTEREST STATEMENT

The authors declare no conflicts of interest.

## ETHICS STATEMENT

Ethics approval was obtained by the Hollywood Private Hospital (HPH382). Informed and written consent was obtained from all individual participants included in the study.

## Data Availability

Data has not been made publicly available, though data sets generated during the current study can be made available from the corresponding author on reasonable request.
